# Hospital implementation of minimally invasive autopsy: A prospective cohort study of clinical performance and costs

**DOI:** 10.1371/journal.pone.0219291

**Published:** 2019-07-16

**Authors:** Ivo M. Wagensveld, M. G. Myriam Hunink, Piotr A. Wielopolski, Folkert J. van Kemenade, Gabriel P. Krestin, Britt M. Blokker, J. Wolter Oosterhuis, Annick C. Weustink

**Affiliations:** 1 Department of Radiology and Nuclear Medicine, Erasmus MC University Medical Center, Rotterdam, The Netherlands; 2 Department of Pathology, Erasmus MC University Medical Center, Rotterdam, The Netherlands; 3 Department of Clinical Epidemiology, Erasmus MC University Medical Center, Rotterdam, The Netherlands; 4 Centre for Health Decision Science, Harvard T.H. Chan School of Public Health, Harvard University, Boston, United States of America; University of British Columbia, CANADA

## Abstract

**Objectives:**

Autopsy rates worldwide have dropped significantly over the last decades and imaging-based autopsies are increasingly used as an alternative to conventional autopsy. Our aim was to evaluate the clinical performance and cost of minimally invasive autopsy.

**Methods:**

This study was part of a prospective cohort study evaluating a newly implemented minimally invasive autopsy consisting of MRI, CT, and biopsies. We calculated diagnostic yield and clinical utility—defined as the percentage successfully answered clinical questions—of minimally invasive autopsy. We performed minimally invasive autopsy in 46 deceased (30 men, 16 women; mean age 62.9±17.5, min-max: 18–91).

**Results:**

Ninety-six major diagnoses were found with the minimally invasive autopsy of which 47/96 (49.0%) were new diagnoses. CT found 65/96 (67.7%) major diagnoses and MRI found 82/96 (85.4%) major diagnoses. Eighty-four clinical questions were asked in all cases. Seventy-one (84.5%) of these questions could be answered with minimally invasive autopsy. CT successfully answered 34/84 (40.5%) clinical questions; in 23/84 (27.4%) without the need for biopsies, and in 11/84 (13.0%) a biopsy was required. MRI successfully answered 60/84 (71.4%) clinical questions, in 27/84 (32.1%) without the need for biopsies, and in 33/84 (39.8%) a biopsy was required. The mean cost of a minimally invasive autopsy was €1296 including brain biopsies and €1087 without brain biopsies. Mean cost of CT was €187 and of MRI €284.

**Conclusions:**

A minimally invasive autopsy, consisting of CT, MRI and CT-guided biopsies, performs well in answering clinical questions and detecting major diagnoses. However, the diagnostic yield and clinical utility were quite low for postmortem CT and MRI as standalone modalities.

## Introduction

Autopsy rates worldwide have dropped significantly, from rates of up to 50% in 1960s to 0–10% today. [1–3] Both in forensic and clinical medicine, the imaging autopsy is increasingly used as adjunct or alternative to the conventional autopsy. Noninvasive autopsies use CT, MRI, or ultrasound as stand-alone test or combinations of diagnostic tests. [4, 5] A minimally invasive autopsy may include laparoscopy, CT-angiography (CTA) and/or image-guided tissue biopsies. [6–19] The value of the modern imaging autopsy in the hospital setting is now under investigation and first studies show that postmortem MRI and CTA have good performance for establishing the cause of death and related or unrelated diagnoses. [6, 20] In this study, we share our results of the implementation of minimally invasive autopsy procedure in our hospital over a 1-year-period. Because not much is reported yet about the clinical performance and costs of such procedures, our aim was to evaluate diagnostic yield and clinical utility—defined as the percentage of successfully answered clinical questions—and to calculate the cost of minimally invasive autopsy—consisting of MRI, CT, and CT-guided biopsies.

## Methods

### Study design and patients

This prospective cohort study was performed at the Erasmus University Medical Center in Rotterdam, the Netherlands, from September 2016 to December 2017. This study was approved by the Erasmus MC Medical Ethical Committee (file number MEC-2011-055). Written informed consent from relatives was acquired in all included cases. During this period both minimally invasive, and conventional autopsy were available to the next-of-kin of all deceased adult patients. Consent for both autopsy methods was asked by the treating physician. In difficult cases we offered the option to perform both minimally invasive autopsy and conventional autopsy, or minimally invasive autopsy combined with a partial conventional autopsy of a specified organ or organ system (e.g. minimally invasive autopsy combined with a partial conventional autopsy of the heart and lungs). In these cases, the minimally invasive autopsy was performed first, and if imaging alone did not answer all clinical questions and give a definite cause of death, minimally invasive autopsy was followed by conventional autopsy, or partial autopsy on the same day. The size of the cohort was determined by the 1-year inclusion period.

### Minimally invasive autopsy

#### Preparation and transportation

The bodies were stored in a refrigerated environment during the period between arrival in the mortuary and the start of imaging. Prior to minimally invasive autopsy the body received general postmortem care in the mortuary. This consisted of cleaning, photographing and removing metal and implanted medical materials in and around the body (only if there was no clinical suspicion about their correct positioning). Next, the body was placed in an MRI compatible body bag.

#### Imaging

MRI was performed on a 1.5T scanner (Discovery MR450, GE Healthcare, Milwaukee, Wisconsin USA), prior to CT scanning. The body was scanned in supine position and was not moved after initial positioning was completed. MRI acquisition contained T1-weighted and T2-weighted scans from head and torso, scanned in 5 segments of 20 cm (Table 1), supplemented with additional acquisitions that depended on the clinical questions (S1 Protocol). MRI scanning time was limited to 1 hour, excluding transportation and positioning.

**Table 1:** Postmortem MRI protocol.

	**T1**	**T2**
Repetition time (ms)	3285	9400
Echo time (ms)	10	42
Inversion time (ms)	892	120
Echo-train-length	6	10
NSA	0.5	1.0
Flip angle (°)	160	160
Coil	Body coil	Body coil
Slice width (mm)	5.0	5.0
FOV (mm)	480x288	480x288
Matrix	384x224	288x160

MRI was performed with a 1.5-T scanner (Discovery MR450; GE Medical Systems, Milwaukee, Wis). NSA = number of signals averaged, FOV = field of view.

CT scans were acquired from head to feet (SOMATOM Definition Edge, Siemens Healthcare, Forchheim, Germany), according to a standardized protocol (Table 2).

**Table 2:** Postmortem CT protocol.

	**Total body** **protocol**	**Head–Neck** **protocol**
Rotation time (s)	1.0	1.0
Tube voltage (kV)	120	100
Tube current (eff. mAs)	400	750
Slice collimation (mm)	2 x 64 x 0.6	2 x 64 x 0.6
Pitch	0.65	0.35
Scan time (s)	69.9	24.1
Reconstruction	Iterative	Iterative

CT was performed with a dual-source CT scanner (SOMATOM Definition Edge; Siemens, Forchheim, Germany) and included scans of brain, neck, torso, and lower extremities.

A board-certified radiologist interpreted the CT and MRI scans directly after acquisition was completed, compared the postmortem scans with available premortem imaging, and identified suspected pathological lesions to plan CT-guided biopsies. The radiologist was familiar with the medical history and had access to the electronic patient record.

#### Biopsies

A board-certified pathologist and the treating clinician were consulted to discuss imaging findings and plan biopsy targets. CT-guided biopsies were performed with a reusable biopsy instrument (Bard Biopsy Systems, Tempe, USA) directly after the CT scan was completed. Biopsies (12 Gauge) were routinely taken from heart, lungs, liver, kidneys, and spleen. Additional biopsies were taken from suspected pathology at imaging. From every biopsy location 4–6 different needle biopsies were sampled to reduce the risk of sampling error. Microbiology cultures and fluid for cytology were sampled upon indication. Histologic staining (H&E) was performed according to department protocol and upon examination of the pathologist additional pathological stains were performed on indication. If consented to, stereoscopic brain biopsies were planned and executed using a stereoscopic navigation system (Brainlab Kolibri, Brainlab, Munich, Germany). The radiologist performed the brain biopsies in the mortuary directly after the CT-guided biopsies of the torso were completed.

#### Reporting

The radiologist made a standardized radiology report that included both imaging findings and biopsy targets. Postmortem imaging was compared to antemortem imaging when available. Radiologists or pathologists in different subspecialties were consulted when organ-specific expertise was required. The pathologist evaluated the biopsies and discussed the imaging and microscopic findings with the radiologist during interdisciplinary meetings. The pathologist integrated the radiological report in the autopsy report and both pathologist and radiologist authorized the final minimally invasive autopsy report. Both the radiologist and pathologist had knowledge of the patient history and clinical questions and these were always addressed in the final report.

**Fig 1:**
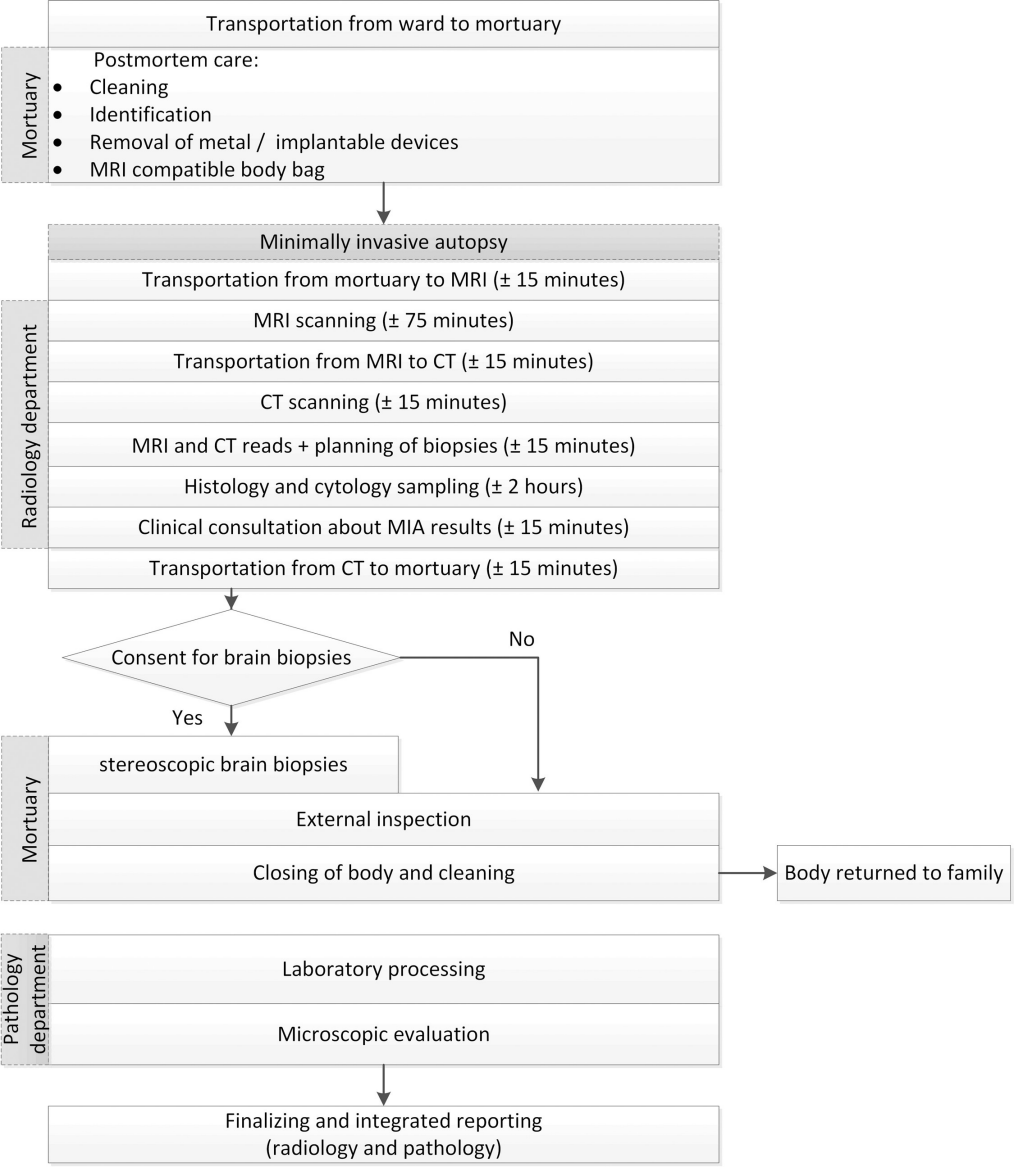
Logistical flow-chart of minimally invasive autopsy.

The whole process of preparation, imaging, biopsies and reporting is schematically shown in Fig 1.

## Data analysis

### Clinical performance

#### Diagnostic yield

All major diagnoses found with minimally invasive autopsy (CT, MRI, biopsies, or partial autopsy) were registered in a clinical data file (Microsoft Excel). Major diagnoses were defined as diagnoses related to the cause of death. For all diagnoses we registered if the diagnosis was found with CT and/or MRI, and if a biopsy was needed. For analysis purposes imaging confidence scores were attributed on the diagnosis level for CT and MRI separately. A diagnosis was scored as 0 if it was found on neither CT nor MRI, a diagnosis was scored as 1 if it was detected on CT or MRI but no likely diagnosis could be made on imaging alone and a biopsy was needed for further elucidation, a diagnosis was scored as 2 if it was detected on imaging with high likelihood of being the suspected diagnosis, but a biopsy is needed for confirmation, and a score of 3 was given if a definitive diagnosis could be made based on CT or MRI and no biopsy was required for confirmation. A Chi-squared test was used to test if there was statistically significant difference in diagnostic yield between CT and MRI.

#### Clinical utility

We calculated the percentage of clinical questions that could be answered by minimally invasive autopsy. To evaluate this, we registered all clinical questions from the minimally invasive autopsy request forms provided by the treating physicians. We calculated the percentage of successfully answered clinical questions. All analyses were performed on the individual question level. We retrospectively evaluated if the radiologist was able to provide an answer to the clinical question with CT or MRI, or the combination of CT and MRI, and in how many cases a biopsy was needed to answer the question. A Chi-squared test was used to test if there was a statistically significant difference between clinical utility of CT and MRI.

### Cost calculation

We evaluated the mean cost per procedure (in Euros) of the minimally invasive autopsy from the perspective of the hospital. We recorded all direct costs of the minimally invasive autopsy including materials used, personnel involved, energy usage, maintenance and depreciation of scanning equipment. Overhead costs were also included in the cost calculation and included the cost of scanning rooms used and planning costs for the imaging procedures. Other costs were use of the hospital’s Picture Archiving and Communication System (PACS), as well as reporting fees. Costs of the pathology department mainly comprised personnel and overhead costs and fees for the histological processing of biopsies. Personnel cost was estimated based on the average wage multiplied by the average amount of time required for the specified parts of the procedure. Cost of cytology and toxicology were not factored in the cost calculation, because both were only performed in a small number of cases. For comparison purposes we also calculated the costs of conventional autopsy as performed in our hospital.

## Results

### Recruitment

**Fig 2:**
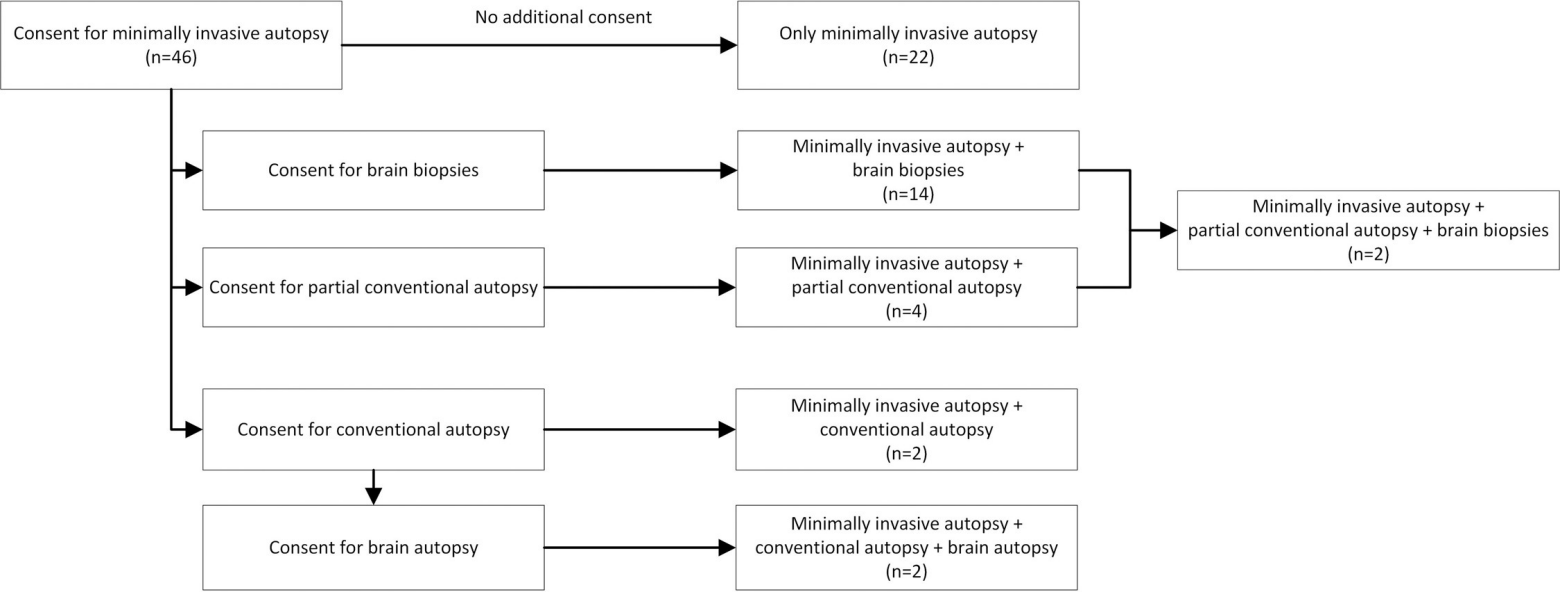
Case inclusion flow-chart.

We evaluated 46 cases that underwent minimally invasive autopsy. In 16 of these cases permission was given for brain biopsies. Within the group with permission for minimally invasive autopsy, there were 6 cases with additional permission for a partial autopsy (in 5 cases partial autopsy of heart and lungs, in 1 case partial autopsy of an adrenal lesion that was seen prior to death). In an additional 4 cases there was permission for a full conventional autopsy after the minimally invasive autopsy, and in 2 of these cases there was also permission for brain autopsy. A case inclusion diagram is shown in Fig 2.

The cohort consisted of 30/46 (65.2%) men and 16/46 (34.8%) women, mean age was 62.9 years old (SD: 17.5, min-max: 18–91). MRI acquisition time was approximately 60 minutes, CT acquisition took 20 minutes including the multiplanar reconstructions. Torso biopsies took between 1.5 and 2.5 hours and brain biopsies approximately 1 hour. The full procedure time including preparation and transportation was 4 to 5 hours on average, depending on the number and location of biopsies necessary. Per minimally invasive autopsy case, 17 different targets were biopsied on average. Cytology samples were taken in 11 cases.

### Clinical performance

#### Diagnostic yield

Ninety-six major diagnoses were found with the minimally invasive autopsy: 47/96 (49.0%) were new diagnoses and in another 15/96 (15.6%) the minimally invasive autopsy revealed an unexpected but clinically relevant diagnosis that was not known prior to death (e.g. new information on the size or etiology of a known malignancy).

MRI had a significantly higher diagnostic yield than CT: 82 were (85.4%) found with MRI and 65 with CT (67.7%) (p = 0.008).

**Fig 3:**
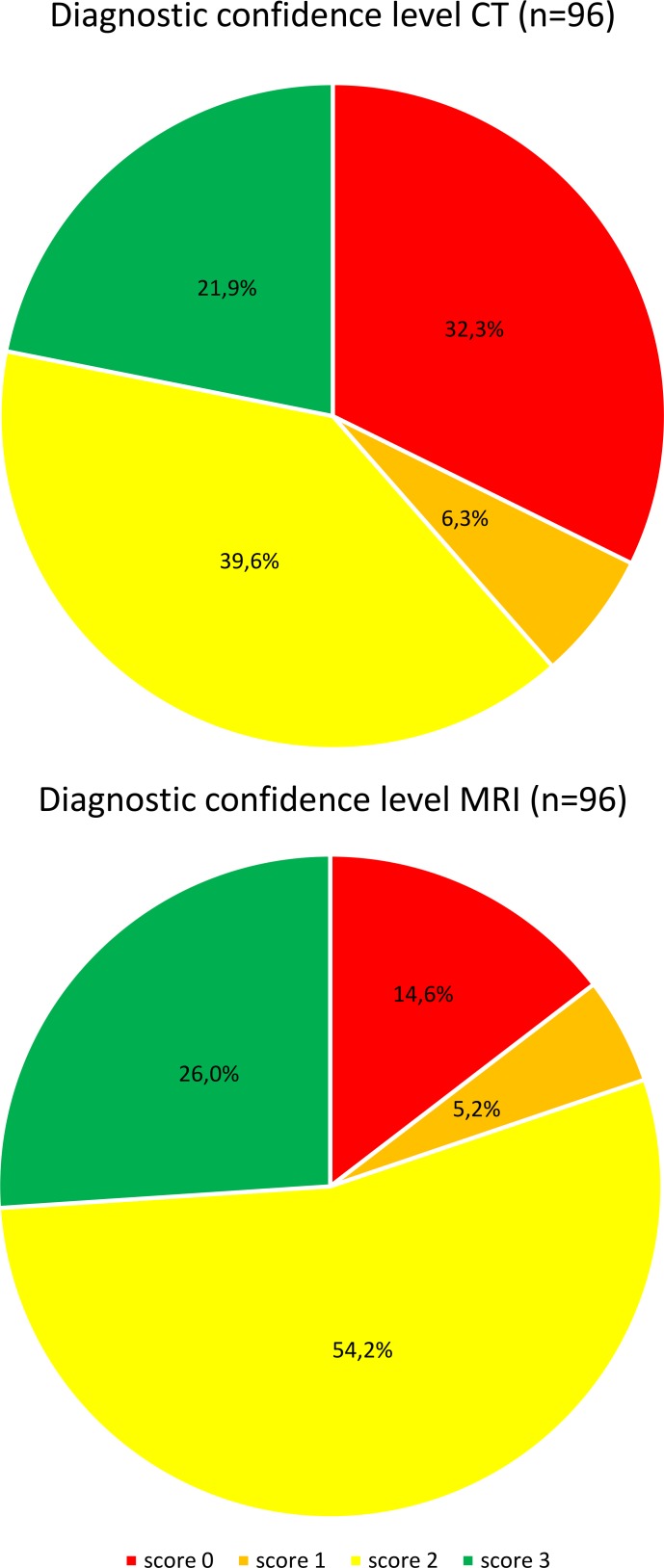
Diagnostic yield of postmortem CT and MRI. *Red–Imaging confidence score 0*: *diagnosis not detected; Orange–score 1*: diagnosis detected on imaging, but unclear; biopsy required; *Yellow–score 2*: *diagnosis seen on imaging and likely*, *biopsy required for confirmation; Green—score 3*: *diagnosis certain*, *no biopsy required*.

**Fig 4:**
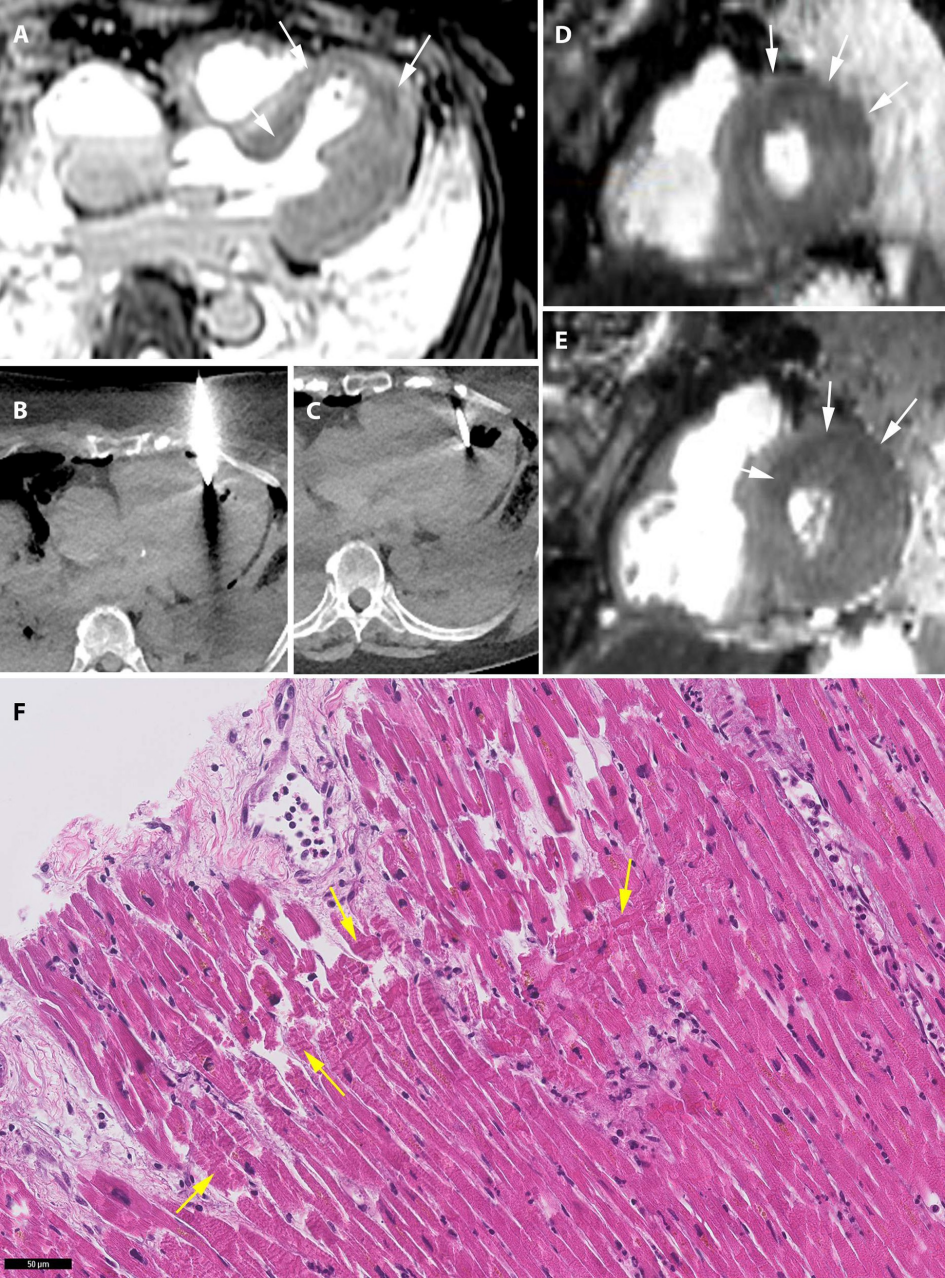
Acute myocardial infarction. 77-year-old woman with a history of hypertension and cerebral ischemia. She was resuscitated unsuccessfully after she was found gasping and unresponsive in bed. Postmortem MRI found T2 hypointensity in the septal, anterior and lateral myocardium (A, D, E: arrows) indicative of acute myocardial infarction in the area supplied by the left anterior descending (LAD) artery. CT-guided biopsies (B and C) from the myocardium were taken and histology showed contraction band necrosis (arrows) confirming acute myocardial infarction (F).

**Fig 5:**
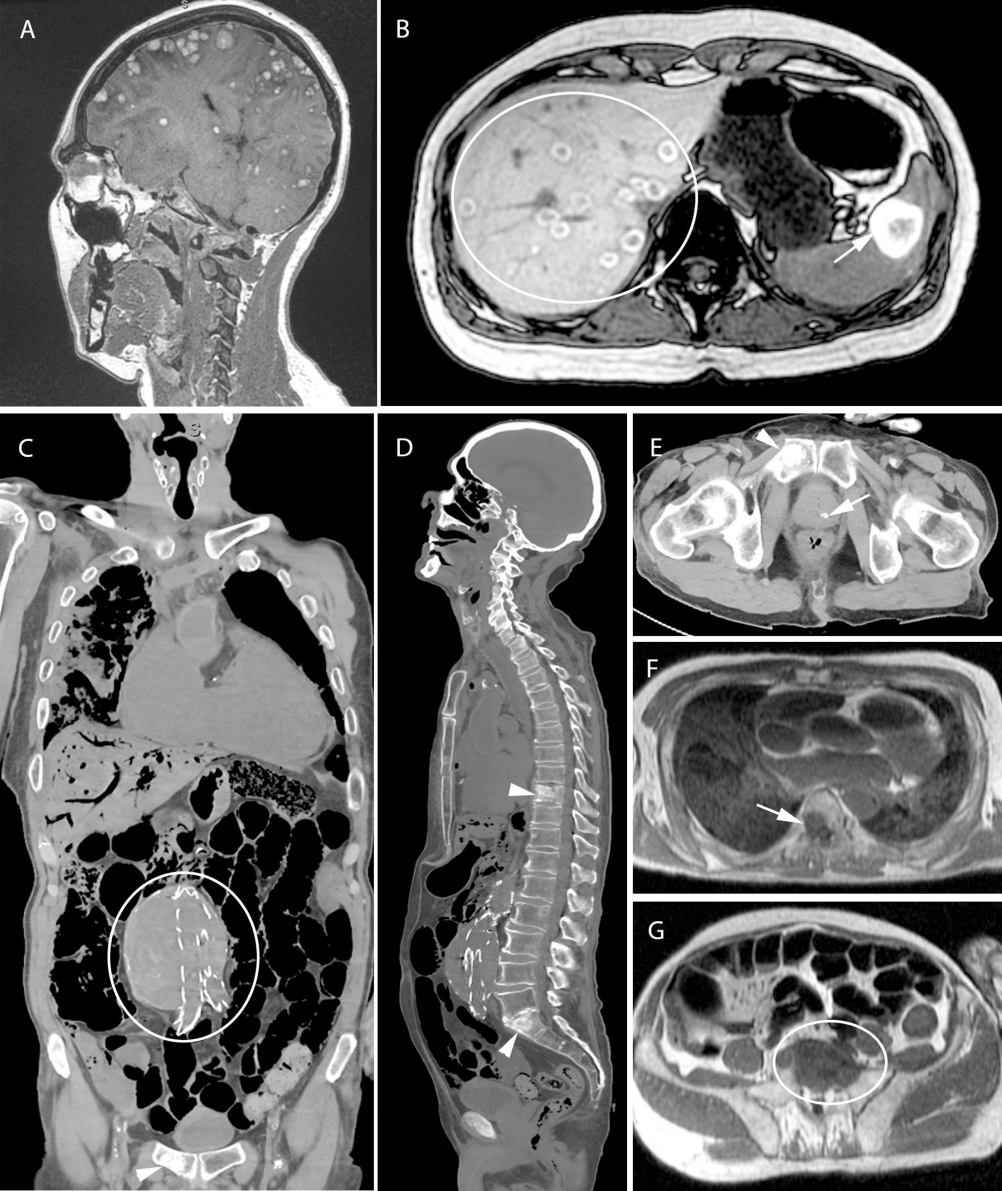
Oncologic cases. A and B: 45-year-old woman with metastasized melanoma. Postmortem T1w MRI shows extensive metastases in brain (A), liver (circle) and spleen (arrows) (B). Tissue was sampled for histologic examination and genetic testing. C through G: 87-year-old man with a known aneurysm of the abdominal aorta, for which he underwent endovascular aortic repair (C: circle). The clinician wanted to exclude aortic rupture or acute myocardial infarction. Minimally invasive autopsy found prostate cancer (E: arrow) with multiple osseous metastases (D and E: arrowheads, F: arrow, G: circle) as unexpected findings. Focal signal abnormalities in the myocardium and histology confirmed acute myocardial infarction as the cause of death (not shown in this image).

**Fig 6:**
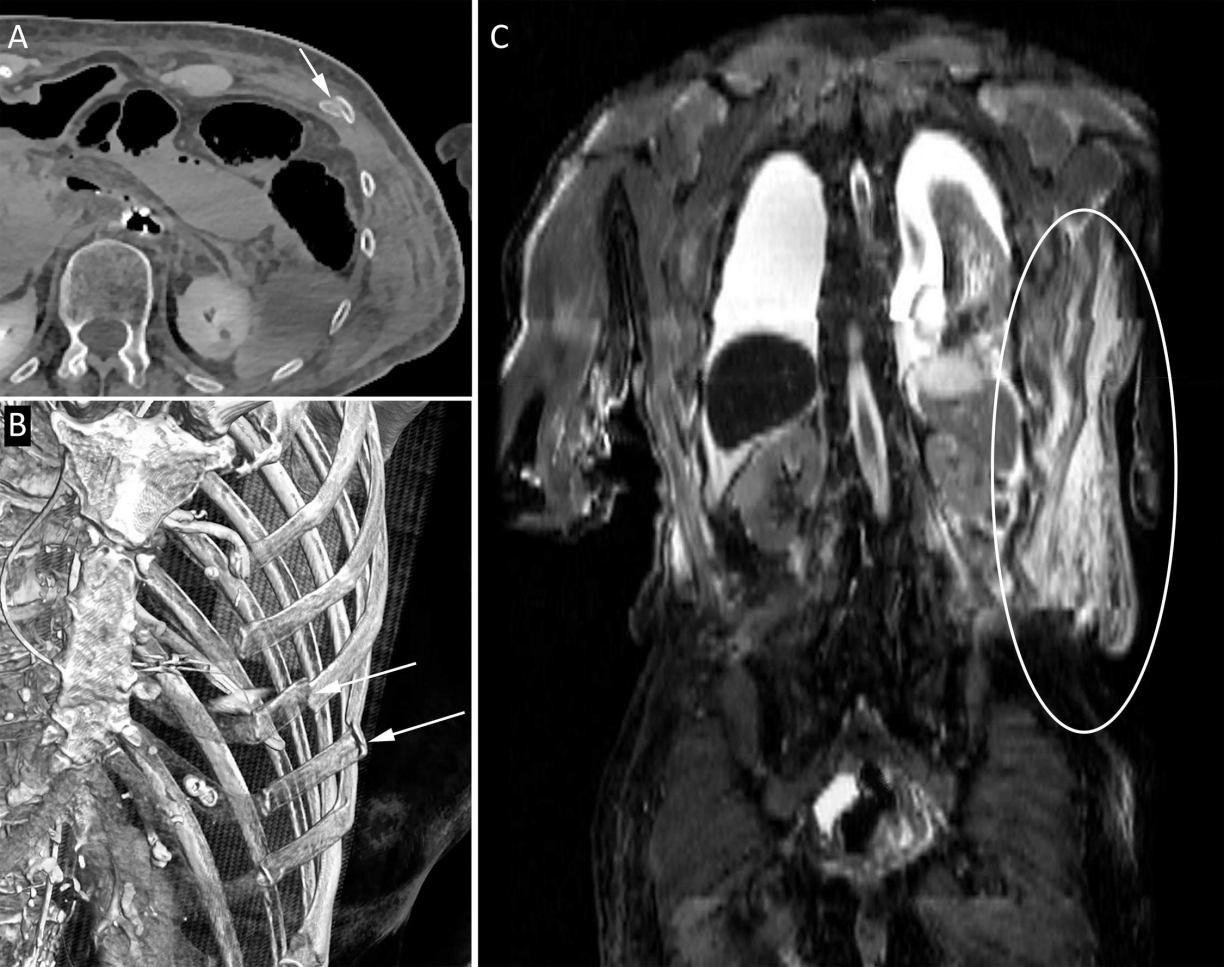
Rib fractures with soft-tissue hematoma. 89-year-old woman who underwent an elective coronary angiography for mitral valve insufficiency and complaints of angina. During the procedure she developed cardiac arrest and required cardiopulmonary resuscitation. She was transported to the intensive care, where she showed symptoms of hypovolemic shock and she died shortly afterwards. Postmortem CT showed multiple rib fractures (A and B: arrows) and on postmortem MRI a large soft-tissue hematoma was seen in the left flank (C: ellipse).

The imaging confidence scores for CT and MRI are shown in Fig 3 and case examples are shown in Figs 4–6. The specific diagnoses per case and corresponding imaging confidence scores are detailed in Table 3.

**Table 3:** Case table.

	Diagn. No.	Diagnosis	Organ	CT confidence	MRI confidence
1	1	Brain herniation	Brain	3	3
	2	Acute myocardial infarction	Heart	0	2
2	1	Chronic myocardial infarction	Heart	0	2
	2	Acute myocardial infarction	Heart	0	2
	3	Pulmonary sequester	Lung	2	2
3	1	Acute myocardial infarction	Heart	0	2
	2	Aspiration	Lung	2	2
4	1	Brain hemorrhage	Brain	3	3
	2	Adrenal hemorrhage	Adrenal gland	2	2
5	1	Lymphoma	Lung	3	3
	2	Aspiration	Lung	1	1
	3	Cellular rejection heart transplant	Heart	0	0
6	1	Chronic myocardial infarction	Heart	0	0
	2	Acute myocardial infarction	Heart	0	0
	3	Brain microbleeds	Brain	0	3
	4	Brain hemorrhage	Brain	2	2
	5	Systemic infection	Brain	0	0
7	1	Encephalitis	Brain	0	0
	2	Systemic metastases	Lung	2	2
	3	Lung tumor	Lung	0	0
8	1	Gastrointestinal tract leakage	Intestines	2	2
	2	Pneumonia	Lung	2	2
9	1	Myocardial hypertrophy	Heart	2	2
	2	Pulmonary edema	Lung	1	1
	3	Encephalitis	Brain	0	0
10	1	Pulmonary embolism	Lung	1	3
11	1	Pneumonia	Lung	2	2
	2	Kidney abscess	Kidney	2	2
12	1	No major diagnosis found	-	-	-
13	1	Chronic myocardial infarction	Heart	2	3
	2	Pulmonary edema	Lung	2	2
14	1	Pneumonia	Lung	2	2
	2	Myocardial hypertrophy	Heart	2	2
	3	Pneumothorax with hematothorax	Lung	3	3
15	1	Acute myocardial infarction	Heart	0	2
	2	Pulmonary edema	Lung	2	2
16	1	Systemic metastases	Bone	2	2
	2	Acute myocardial infarction	Heart	0	2
	3	Prostate cancer	Prostate	2	2
17	1	Systemic metastases	Systemic	3	3
18	1	Brain hemorrhage	Brain	3	3
	2	Pneumothorax	Lung	3	3
	3	Pneumonia	Lung	2	2
	4	Muscle dystrophy	Muscle	3	3
19	1	Rupture of abdominal aneurysm	Vascular	3	3
20	1	Acute myocardial infarction	Heart	0	2
21	1	Gastrointestinal tract bleeding	Intestines	2	2
22	1	Cardiomyopathy	Heart	2	2
	2	Pneumonia	Lung	2	2
	3	Acute myocardial infarction	Heart	0	2
23	1	Systemic iron overload	Systemic	0	3
	2	Gastrointestinal tract infection	Intestines	0	2
24	1	Acute myocardial infarction	Heart	0	2
25	1	Ischemia	Intestines	2	2
	2	Cardiomyopathy	Heart	2	2
26	1	Heart failure	Heart	2	2
	2	Focal liver steatosis	Liver	2	2
27	1	Aspergillus infection	Lung	2	2
	2	Graft-versus-host-disease	Systemic	0	0
28	1	Lung tumor	Lung	3	3
	2	Systemic metastases	Liver	2	2
	3	Aspiration	Lung	1	1
29	1	Acute myocardial infarction	Heart	0	2
30	1	Systemic metastases	Systemic	3	3
31	1	Pneumonia	Lung	2	2
	2	Lymphoma	Systemic	3	3
32	1	Aspiration	Lung	2	2
	2	Acute pancreatitis	Pancreas	2	2
33	1	Hematothorax	Lung	3	3
	2	Pneumothorax	Lung	3	3
	3	Rib fractures	Skeleton	3	2
	4	Pulmonary hypertension	Lung	2	2
	5	Spondylodiscitis	Skeleton	2	2
34	1	Liver cirrhosis	Liver	3	3
	2	Acute tubular necrosis	Kidney	0	0
35	1	Acute myocardial infarction	Heart	0	0
	2	Pancreatic carcinoma	Pancreas	2	2
36	1	Acute myocardial infarction	Heart	0	2
37	1	Pulmonary embolism	Lung	1	3
	2	Chronic myocardial infarction	Heart	0	1
38	1	Intestinal wall thickening (inflammation)	Intestines	1	1
39	1	Rupture of abdominal aneurysm	Vascular	3	3
	2	Breast cancer	Breast	2	2
40	1	Acute myeloid lymphoma	Systemic	0	2
	2	Leukostasis	Lung	2	2
	3	Systemic infection	Systemic	0	0
41	1	Pulmonary fibrosis	Lung	0	0
	2	Hematothorax	Lung	3	3
	3	Acute pneumonia	Lung	0	0
42	1	Hematothorax	Lung	3	3
43	1	Chronic myocardial infarction	Heart	0	2
44	1	Retroperitoneal hematoma	Soft tissue	3	3
	2	Thoracic wall hematoma	Soft tissue	3	3
45	1	Aspiration	Lung	2	2
46	1	Ileus	Intestines	2	2
	2	Intestinal ischemia	Intestines	2	2
	3	Acute tubular necrosis	Kidney	0	0

#### Clinical utility

A total of 84 clinical questions were asked in 46 minimally invasive autopsy procedures. Seventy-one (84.5%) of these questions could be answered with minimally invasive autopsy.

CT could answer 34/84 (40.5%) of clinical questions; in 23/84 (27.4%) without the need for biopsies, and in 11/84 (13.0%) a biopsy was required. MRI could answer 60/84 (71.4%) of clinical questions, in 27/84 (32.1%) without the need for biopsies, and in 33/84 (39.8%) a biopsy was required. MRI had significantly higher clinical utility than CT (p<0.001).

The combination of CT and MRI could answer 65/84 (77.4%) of clinical questions, in 30/84 (35.7%) without the need for biopsies, and in 35/84 (41.7%) a biopsy was required. Six additional questions of the 84 (7.1%) were answered based on histological findings that were not seen on imaging (i.e. biopsies taken with random sampling of organs). Table 4 shows the performance of CT and MRI for clinical questions in our cohort and gives an estimate of the hypothetical performance, based on expert opinion, of conventional autopsy for these questions.

**Table 4:** Clinical utility of postmortem CT and postmortem MRI for specific clinical questions.

Clinical question	n	CT performance	MRI performance	Biopsy required	Hypothetical performance of conventional autopsy
Brain hemorrhage	3	Good	Good	No	Good
Brain ischemia	4	Fair	Good	Yes	Good
Meningitis	1	Poor	Poor	Yes	Good
Myocardial infarction	13	Poor	Good	Yes	Good
Cardiomyopathies	2	Poor	Fair	Yes	Good
Coronary artery disease	1	Fair	Poor	No	Good
Cardiac arrythmia	2	Poor	Poor	No	Fair
Pulmonary embolism	6	Poor	Fair	Yes	Good
Pneumonia	3	Fair	Fair	Yes	Good
Pulmonary hemorrhage / Hemothorax	3	Fair	Fair	No	Good
Rupture of abdominal aortic aneurysm	2	Good	Good	No	Good
Intestinal perforation	1	Fair	Fair	No	Good
Intestinal ischemia	2	Fair	Fair	Yes	Good
Liver cirrhosis	2	Fair	Good	Yes	Good
Implanted organ rejection / graft-versus-host disease	2	Poor	Poor	Yes	Good
Status of (oncological) disease	4	Good	Good	No	Fair
Position of implanted devices	3	Good	Good	No	Fair
Infection focus	4	Fair	Fair	Yes	Fair

CT and MRI performance were based on expert opinion of the postmortem radiologist (ACW). Hypothetical performance of conventional autopsy was based on expert opinion of the pathologist (JWO).

### Costs

The mean cost of a minimally invasive autopsy was €1296 including brain biopsies and €1087 without brain biopsies. Mean cost of CT was €117 and of MRI €215. Mean cost of CT-guided biopsies was €685. The different components of minimally invasive autopsy and their respective cost are detailed in Table 5.

**Table 5:** Costs of minimally invasive autopsy.

	Postmortem care & external inspection	MRI	CT	CT-guided biopsies	Brain biopsies
**Personnel**	Mortuary personnel	Students	Students	Students	Radiologist
	Pathology resident	Radiographer	Radiographer	Radiographer	Mortuary personnel
	Pathologist	Radiologist	Radiologist	Radiologist	
				Pathologist	
**Mean cost (€)**	15	109	67	240	115
**Materials**	MRI compatible body bag	Cleaning materials	Cleaning materials	Protective clothing	Cleaning materials
	Protective clothing	Protective clothing	Protective clothing	Cleaning materials	Protective clothing
	Other materials			Biopsy gun + needle	Drill
				Other materials	
**Mean cost (€)**	55	3	3	40	3
**Equipment**	-	Scanner depreciation	Scanner depreciation	Scanner depreciation	Brainlab navigation
		Power usage	Power usage	Power usage	Maintenance
		Maintenance	Maintenance	Maintenance	Neurosurgical skull clamp
**Mean cost (€)**		40	16	23	15
**Other costs**	-	Overhead	Overhead	Overhead	Overhead
		Reporting	Reporting	Reporting	Reporting
		PACS	PACS	PACS	Histology processing
				Histology processing	
**Mean cost (€)**		63	31	382	76
**Total mean cost (€)**	70	215	117	685	209

PACS = picture archiving and communication system.

The mean cost of a full conventional autopsy was €991 including brain autopsy and €740 without brain autopsy. Costs of the different components of conventional autopsy are found in Table 6.

**Table 6:** Costs of conventional autopsy.

	Postmortem care	External inspection	Dissection	Microscopy	Brain autopsy	Reporting
**Personnel**	Mortuary personnel	Pathology resident	Mortuary personnel	Pathology resident	Laboratory assistant	Pathology resident
		Pathologist	Pathology resident	Pathologist	Mortuary personnel	Pathologist
		Mortuary personnel	Pathologist	Mortuary personnel		
**Materials**	Protective clothing	Protective clothing	Protective clothing	Protective clothing	Protective clothing	**-**
	Other materials		Cleaning materials	Other materials	Cleaning materials	
			Other materials		Other materials	
**Equipment**	**-**	-	Autopsy table	Autopsy table	Autopsy table	-
**Other costs**	Overhead	Overhead	Overhead	Overhead	Overhead	Overhead
				Histology processing	Histology processing	
**Cost (€)**	50	15	142	419	251	114

## Discussion

This study on a minimally invasive autopsy, using MRI, CT and CT-guided biopsies, provides insight into the diagnostic yield, clinical utility and costs of commonly used postmortem imaging methods. The percentage of clinical questions answered with the complete minimally invasive autopsy method (MRI, CT and biopsies combined) was very high (84.5%), but CT and MRI as standalone modalities left many questions unanswered. Diagnostic yield of MRI was higher than CT: 85% of diagnoses were found with MRI versus 68% with CT. Most diagnoses required biopsies for confirmation; diagnostic yield of CT and MRI without biopsies was low. The mean cost of a full minimally invasive autopsy was €1296, and by far the most expensive part of the procedure were the CT-guided biopsies. For comparison, a full conventional autopsy (including brain autopsy) in our hospital had a mean cost of €991. In terms of time; a minimally invasive autopsy took approximately 4 to 5 hours, depending on the complexity of the case and the required biopsies. A conventional autopsy on average takes between 2.5 and 4 hours.

In a previously published study, we compared the performance for finding the immediate cause of death and related major diagnoses of minimally invasive autopsy—consisting of CT, MRI and CT-guided needle biopsies–versus a conventional autopsy. Minimally invasive autopsy and conventional autopsy performed equally well in finding the cause of death, whereas minimally invasive autopsy found a greater number of major diagnoses than conventional autopsy. Furthermore, both minimally invasive autopsy and conventional autopsy found unexpected postmortem findings that were considered relevant new information for the treating physicians: 124/288 (43.1%) of the major diagnoses and 17/99 (17%) of the causes of death were not clinically suspected before death. [21]

Earlier studies on the diagnostic performance of non-invasive and minimally invasive autopsies found that the performance of contrast enhanced imaging methods was better than that of non-contrast enhanced imaging. Furthermore, methods combining radiology with tissue-biopsies had a higher diagnostic performance than imaging alone. [11, 17]

In this study we evaluated the clinical utility of minimally invasive autopsy, defined as the percentage of successfully answered clinical questions by this new autopsy method. In our experience this outcome measure is highly valued by clinicians when they request an autopsy, and often this is considered equally as important as finding the cause of death. Our results show that the diagnostic yield and clinical utility of CT and MRI without biopsy is low. This finding is in line with diagnostic studies on imaging-based autopsies: methods combining imaging with biopsy showed highest sensitivity and specificity. [11, 21] Nevertheless CT and MRI both have strengths and weaknesses. CT is noninvasive, relatively cheap, and widely available. Additionally, CT performs well at visualizing (abnormal) air collections and skeletal abnormalities, such as fractures and bone lesions. A clinical study found that postmortem CT is more accurate in establishing the cause of death than the clinician. [22] However, our results indicate a low performance of CT as standalone test, because unenhanced CT lacks the high soft-tissue contrast necessary to diagnose some common causes of death, such as acute myocardial infarction. [23] Studies on postmortem CTA show that it has a higher sensitivity, but CTA is logistically challenging to perform in a clinical setting and is not purely non-invasive. [24] MRI has excellent soft-tissue contrast, but it is more expensive and generally has longer scanning times and lower availability than unenhanced CT.

Because of its good soft-tissue discrimination, MRI was able to detect more major diagnoses in our study cohort. Cardiac diagnoses in particular were better diagnosed with MRI; acute myocardial infarctions were not seen at all with unenhanced CT-imaging, but MRI detected 10 out of 12 myocardial infarctions. Chronic myocardial infarction can sometimes be detected with CT in a late stage where wall thinning is present, in our cohort this was seen in 1 case. MRI also performed much better than CT in detecting chronic myocardial infarctions: chronic infarctions were detected with MRI in 4 out of 5 cases, and with CT in only 1 out of 5 cases. [25] Pneumonia, another common cause of death, was detected both with MRI and CT, but postmortem artefacts in the lungs such as internal livores can mask or mimic pathologic processes and therefore biopsies of lung abnormalities are always recommended. [26]

A closer look at the performance for answering clinical questions of CT and MRI (Table 4) shows that CT and MRI can answer most questions, but biopsies are still necessary in the majority of questions. Intestinal ischemia and perforation, as well as organ transplant rejection and graft-versus-host disease are difficult to diagnose on imaging. Also, finding the etiology of cardiac arrythmia’s is almost impossible with CT and MRI. For these diagnoses it is recommended to perform a conventional autopsy when consent can be obtained. Minimally invasive autopsy outperforms the conventional autopsy in cases where a full-body assessment is required, e.g. the status or growth of systemic diseases or oncological processes. All clinical questions regarding skeletal lesions or pathology involving air can be answered with postmortem imaging (sometimes combined with biopsies) and in these cases we recommend either a minimally invasive autopsy, or otherwise combining a conventional autopsy with postmortem CT. Furthermore the location of implanted devices is more reliably assessed on imaging, because the position can be seen in-situ, whereas with conventional autopsy artificial movement can occur during the opening of the body or removal of organs.

For our minimally invasive autopsy method we opted for a combination of CT, MRI and CT-guided biopsies, because it combines the soft-tissue contrast of MRI, to screen the brain and torso for soft-tissue abnormalities that can then be targeted with CT-guided biopsies. We designed multiple different MRI protocols for different clinical scenarios. This allowed us to scan with a higher resolution in the region of interest and use specific MRI-sequences when a particular question demanded it, without increasing MRI scanning time to more than 1 hour: e.g. when cerebral microhemorrhages were suspected a susceptibility-weighted MRI sequence would be made of the brain, and when a young adult died suddenly and sudden-cardiac death was suspected a high-resolution cardio-thoracic MRI would be performed.

Other studies have suggested that using postmortem MRI as a screening tool prior to conventional autopsy in selected cases may lead to a cost reduction; in this case postmortem MRI is performed first and is only followed by conventional autopsy if the MRI does not give a definitive cause of death. [27] In a similar fashion we could perform a slimmed down version of the minimally invasive autopsy on a case-per-case basis depending on the clinical questions. For specific questions it may be possible to omit either CT or MRI and take only biopsies of organs or tissues of clinical importance. The risk of missing important diagnoses needs to be considered though; in our cohort 14/96 (14.6%) of diagnoses were not seen on imaging, and would have been missed if no random sampling of all organs was performed. The sampling success rate in our validation study was very high (>95%), because we sampled at least 4 needle biopsies per target location.

Future research should focus on different imaging strategies for various clinical circumstances in which patients die. The cost that a hospital is willing to pay and the measure of invasiveness the next-of-kin are willing to accept are important factors when deciding how to design a postmortem imaging service. These considerations can be different depending on the setting. For example, an intensive care doctor might be more interested in knowing the cause of death and may be less interested in diagnoses already known or unrelated to death. In contrast, a clinical oncologist is more eager to know the exact tumor type in patients who were unresponsive to treatment, warranting a more invasive technique.

Our study has several limitations. Our study includes a minimally invasive autopsy that combines CT, MRI and biopsies. Alternative procedures, such as postmortem CTA, MRA, and ultrasound were not part of our imaging protocol. The addition of CTA could greatly increase the clinical performance of minimally invasive autopsy for coronary heart disease. Our cohort was relatively small, therefore we made no attempt to aggregate diagnoses per organ system. The costs of minimally invasive autopsy were calculated based on direct material, personnel and overhead cost. We did not take into account that the overall cost per individual procedure tends to increase when fewer procedures in total are performed, as overhead costs and wages for personnel are shared between the total amount of procedures. [20] Finally, the population that we scanned is dependent on country and setting; we investigated the performance and costs in an academic hospital in the Netherlands. More studies are necessary to evaluate performance and costs in different settings and countries.

## Conclusions

A minimally invasive autopsy, consisting of CT, MRI and CT-guided biopsies, performs well in answering clinical questions and detecting major diagnoses. However, the diagnostic yield and clinical utility were quite low for postmortem CT and MRI as standalone modalities.

## Supporting information

S1 Protocol Supplementary MRI protocols. (DOCX)
