# Supplementary material for: Hospital implementation of minimally invasive autopsy: A prospective cohort study of clinical performance and costs
Source: PLoS One. 2019 Jul 16;14(7):e0219291. doi: 10.1371/journal.pone.0219291 (PMC6634385; doi:10.1371/journal.pone.0219291)
Supplement: S1 Protocol — (DOCX) [file pone.0219291.s001.docx]

Appendix A: supplementary MRI protocols

## Protocol: abdomen

Coil setup: 12 channel Body large

Body setup: Feet first

| Region | Scans | Coil |
| --- | --- | --- |
| Head | Localizer 1  DWI b0,b1200 | Body coil |
|  |  |  |
| Whole body (standard) | Localizers 2, 3  Flair T1w (5 sections)  IRFSE-STIR-T2w (5 sections) | Body coil |
|  |  |  |
| Abdomen (high resolution) | Asset calibration  IRFSE-STIR-T2w TE40ms | 12 Channel body |
|  | DWI b0, b800  LAVAFlex T1w  Cube T2w TE 180ms |  |

## Protocol: general

Coil setup: Head-neck-spine (HNS)

Body setup: Head first

| Region | Scans | Coil |
| --- | --- | --- |
| Head | Localizer 1  Asset calibration  DWI b0,b1200 | HNS |
|  |  |  |
| Whole body (standard) | Localizers 2,3  S1-S5 FlairT1w  S1-S5 IRFSE-STIR-T2w | Body coil |

## Protocol: Head

Coil setup: Head-neck-spine (HNS)

Body setup: Head first

| Region | Scans | Coil |
| --- | --- | --- |
| Head | Localizer 1  Asset calibration  Sag FSPGR BRAVO T1  Sag CUBE DIR  DWI b0,b1200  Ax SPGR 16e | HNS |
|  |  |  |
| Whole body (standard) | Localizers 2,3  S1-S5 FlairT1w  S1-S5 IRFSE-STIR-T2w | Body coil |

## Protocol: Head-heart

Coil setup: Head-neck-spine (HNS) + Brachial plexus attachment

Body setup: Head first

| Region | Scans | Coil |
| --- | --- | --- |
| Head | Localizer 1  ASSET calibration  Sag FSPGR BRAVO T1  Sag CUBE T2  Sag CUBE DIR  DWI b0,b1200  Ax SPGR 16e | HNS |
|  |  |  |
| Whole body (standard) | Localizers 2,3  S1-S5 FlairT1w  S1-S5 IRFSE-STIR-T2w | Body coil |
|  |  |  |
| Heart | ASSET calibration  Hrt-DWI-b0, b1000 | HNS brachial plexus |

## Protocol: Head, Heart & Thorax

Coil setup: Head-neck-spine (HNS) + Brachial plexus attachment

Body setup: Head first

| Region | Scans | Coil |
| --- | --- | --- |
| Head | Localizer 1  ASSET calibration  Sag FSPGR BRAVO T1  Sag CUBE T2  Sag CUBE DIR  DWI b0,b1200  Ax SPGR 16e | HNS |
|  |  |  |
| Whole body (standard) | Localizers 2,3  S1-S5 FlairT1w  S1-S5 IRFSE-STIR-T2w | Body coil |
|  |  |  |
| Heart | ASSET calibration  Hrt-DWI-b0, b1000 | HNS brachial plexus |
|  |  |  |
| Thorax | Trx LAVAFlex T1w  Trx T1w FS  Trx FS Cube T2w 80ms | HNS brachial plexus |

## Protocol: Head, Heart & Thorax

Coil setup: Head-neck-spine (HNS) + Brachial plexus attachment

Body setup: Head first

| Region | Scans | Coil |
| --- | --- | --- |
| Head | Localizer 1  ASSET calibration  Sag FSPGR BRAVO T1  Sag CUBE T2  Sag CUBE DIR  DWI b0,b1200  Ax SPGR 16e | HNS |
|  |  |  |
| Whole body (standard) | Localizers 2,3  S1-S5 FlairT1w  S1-S5 IRFSE-STIR-T2w | Body coil |
|  |  |  |
| Heart | ASSET calibration  Hrt-DWI-b0, b1000 | HNS brachial plexus |
|  |  |  |
| Thorax | Trx LAVAFlex T1w  Trx T1w FS  Trx FS Cube T2w 80ms | HNS brachial plexus |
|  |  |  |
| Abdomen/Pelvis | Ax Abd LAVAFlex T1w  Ax Abd FS Cube T2w 120ms | Body coil |

## Protocol: Oncologic: Thorax-Abdomen

Coil setup: 12 channel body coil

Body setup: Feet first

| Region | Scans | Coil |
| --- | --- | --- |
| Head | Localizer 1  DWI b0,b1200 | Body coil |
|  |  |  |
| Whole body | Localizers 2,3  S1-S5 FlairT1w  S1-S5 IRFSE-STIR-T2w  S1-S7 DWI b0,b100,b1100 | Body coil |
|  |  |  |
| Thorax-abdomen | Body ASSET cal  Body LAVAFlex T1w  Body T1w FS  Body IRFSE-STIR-T2w | 12 channel body |

Note.—MRI was performed with a 1.5-T unit (Discovery MR450; GE Medical Systems, Milwaukee, Wis).

## Protocol: Thorax, Heart

Coil setup: 32 channel cardiac coil

Body setup: Feet first

| Region | Scans | Coil |
| --- | --- | --- |
| Head | Localizer 1  DWI b0,b1200 | Body coil |
|  |  |  |
| Whole body (standard) | Localizers 2,3  S1-S5 FlairT1w  S1-S5 IRFSE-STIR-T2w | Body coil |
|  |  |  |
| Thorax | ASSET calibration  Trx IRFSE-STIR-T2w  Trx LAVAFlex T1w  Trx T1w FS | 32 channel cardiac |
|  |  |  |
| Heart | Hrt-DWI-b0, b1000 | 32 channel cardiac |
|  |  |  |
| Coronaries | Corn LavaFlex T1w | 32 channel cardiac |
